# Real-space visualization of order-disorder transition in BaTiO_3_

**DOI:** 10.1126/sciadv.adx9804

**Published:** 2025-09-03

**Authors:** Yang Zhang, Xiaoming Shi, Suk Hyun Sung, Cong Li, Houbing Huang, Pu Yu, Ismail El Baggari

**Affiliations:** ^1^ The Rowland Institute at Harvard, Harvard University, Cambridge, MA 02138, USA.; ^2^ Department of Physics, University of Science and Technology Beijing, Beijing, China.; ^3^ State Key Laboratory of Low Dimensional Quantum Physics and Department of Physics, Tsinghua University, Beijing, China.; ^4^ Advanced Research institute of Multidisciplinary Science, and School of Materials Science and engineering, Beijing institute of Technology, Beijing 100081, China.

## Abstract

Ferroelectricity in BaTiO_3_ was observed nearly 80 years ago, but the mechanism underlying its ferroelectric-paraelectric phase transition remains elusive. The order-disorder transition has been recognized as playing a critical role; however, the precise nature of the order parameter still remains under scrutiny, including the local dipole direction and the correlations above and below the Curie temperature. Using in situ scanning transmission electron microscopy, we directly map polar displacements in BaTiO_3_ across the ferroelectric-paraelectric phase transition, providing atomistic insights into an order-disorder mechanism. Atomic tracking reveals finite polar Ti displacements in the paraelectric phase where they manifest as random polar nanoregions. The displacements align along <111> direction in both the ferroelectric and paraelectric phases. The paraelectric-ferroelectric transition emerges from real-space correlations of the <111> polar Ti displacements. Our direct visualizations provide atomic insights into the order-disorder mechanism in the ferroelectric-paraelectric transition of BaTiO_3_.

## Teaser

Real-space visualization provides atomic insight into order-disorder transition in BaTiO_3_.

## INTRODUCTION

Structural phase transitions in materials involve changes in symmetry that are tied to functional properties. The simplest structural phase transition involves a displacive mechanism, whereby a softening displacement mode (phonon) freezes into a static pattern. An alternative mechanism is order-disorder (O-D), where the transition involves the evolution of correlations of preformed clusters of order. Ferroelectric (FE) phase transitions often follow one of these mechanisms (*1*). For instance, the phase transition of PbTiO_3_ is associated with the coherent displacement of Ti and Pb atoms along <100> relative to their positions in the high-symmetry phase (*2*). Conversely, NaNO_2_ undergoes a phase transition involving an emergent ordering of N^3+^ and O^2−^ ions, generating long-range dipoles in the FE phase (*3*).

**Fig. 1.**
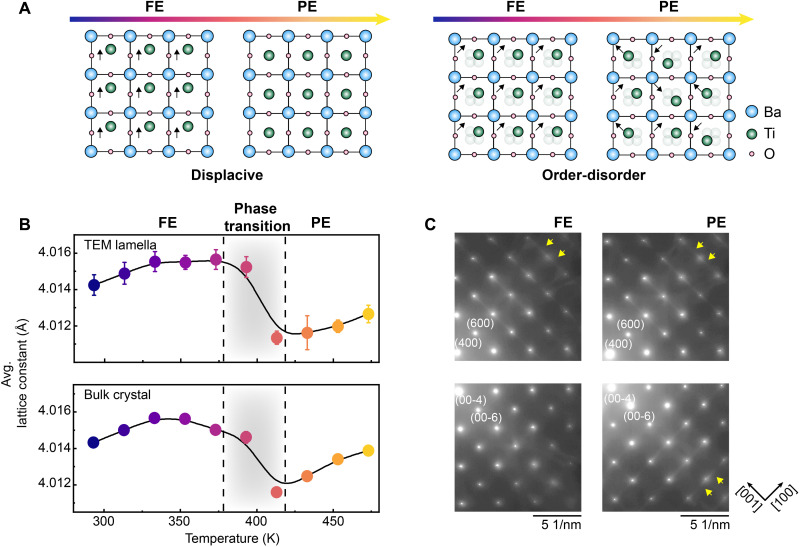
FE-PE phase transition of BaTiO_3_ reproduced by in situ STEM. (**A**) Schematic graphs showing the displacive model (left) and O-D model (right) of the FE-PE phase transition. The arrow represents the Ti displacement. The transparent atom highlights the occupational distribution in O-D case. (**B**) Averaged lattice constant measured from a single domain in the ADF-STEM image of TEM lamella (top) and XRD (bottom) of bulk crystal at different temperatures. The error bar comes from the mean absolute error of the normal distribution fitting. The dashed line and gray shadow determine the phase transition region. (**C**) Zoom-in diffraction patterns collected from FE (left) and PE (right) phases. The projection is the [010] zone axis. The yellow arrows highlight the diffuse intensity.

BaTiO_3_ is the first pervoskite transition-metal oxide identified to exhibit ferroelectricity (*4*), which originates from the off-center Ti displacements relative to the center of TiO_6_ octahedron (**Δ**_Ti_). Owing to its low switching barrier and polymorphic phases, BaTiO_3_ has been extensively studied across a wide range of fields, including logic-in-memory (*5*, *6*), nanogenerators (*7*, *8*), energy storage (*9*, *10*), and photonic devices (*11*, *12*). Despite its robust ferroelectricity and widespread applications, the mechanism underlying the FE-PE phase transition remains elusive. In its bulk crystal form, BaTiO_3_ has a sequence of structural transitions, spanning rhombohedral (<180 K), orthorhombic (180 to 280 K), tetragonal (280 to 390 K), and cubic (>390 K) phases (*13*). The FE–paraelectric (PE) phase transition coincides with the tetragonal-cubic structural transition (the FE and PE correspond to tetragonal and cubic phase, respectively), occurring at the Curie temperature, *T_c_*, of around 393 K (*14*). The displacive model attributes this transition to the absence of **Δ**_Ti_ in the PE phase, where both the global and local dipole moment vanish (*15*–*17*) (left panel of Fig. 1A). Conversely, the O-D model posits that **Δ**_Ti_ persist locally in the PE phase but with no long-range correlations between them (right panel of Fig. 1A) (*18*–*22*). Although the O-D model has been recognized as playing a critical role in BaTiO_3_ through various studies spanning Raman spectroscopy, x-ray scattering, neutron scattering, infrared reflectivity, second harmonic generation, and convergent-beam electron diffraction (*23*–*31*), the precise structure in the PE phase, as well as the evolution of local correlations of polar displacements in real space, remains elusive, persisting as a subject of debate for over 50 years (*18*, *24*, *25*, *32*–*36*).

Here, we present real-space visualizations of FE order parameters across the FE-PE phase transition in BaTiO_3_, uncovering the atomistic mechanism underlying the O-D model. Using in situ scanning transmission electron microscopy (in situ STEM), we measure finite **Δ**_Ti_ that persists in the PE phase, determine the <111> direction of **Δ**_Ti_, and elucidate an evolution of the real-space correlations of **Δ**_Ti_ across the PE-FE transition. Our atomic tracking provides critical insights into the phase transition within the framework of the O-D model in BaTiO_3_. Furthermore, it establishes a direct link between real-space correlations and macroscopic signatures such as diffuse lines observed in reciprocal space studies (*37*–*40*).

## RESULTS

### Finite **Δ**_Ti_ in the PE phase

We first perform measurements of the lattice constant across the FE-PE transition in a thin, electron transparent BaTiO_3_ sample for transmission electron microscopy (TEM). As shown in Fig. 1B, the lattice constant obtained from annular dark-field (ADF)–STEM images exhibits a drop near 390 K, consistent with x-ray diffraction (XRD) measurements of the bulk crystal (bottom panel of Fig. 1B; raw data are shown in fig. S1). The negative thermal expansion coincides with the FE-PE phase transition temperature of BaTiO_3_ (*41*, *42*). The agreement between TEM and XRD demonstrates that the phase transition behavior in the electron transparent sample matches bulk BaTiO_3_.

Across the Curie temperature, the selected-area electron diffraction (SAED) patterns reveal some differences between the FE and PE phases. Figure 1C shows SAED patterns collected along the [010] zone axis of BaTiO_3_ at 293 K (FE) and 473 K (PE), respectively (raw data are shown in fig. S2). In addition to sharp Bragg spots, we observe diffuse intensity in both phases (highlighted by yellow arrows), indicating the presence of correlated disorder (*43*). Diffuse intensity lines are prominent at high index spots, suggesting the contribution from positional disorder instead of chemical disorder (see Supplementary Text and fig. S3 for more details). In the FE phase, diffuse intensity lines are present only along the [001] direction, whereas in the PE phase, they appear along both the [001] and [100] directions, indicating a transition from anisotropic to isotropic local correlations (*37*–*40*).

**Fig. 2.**
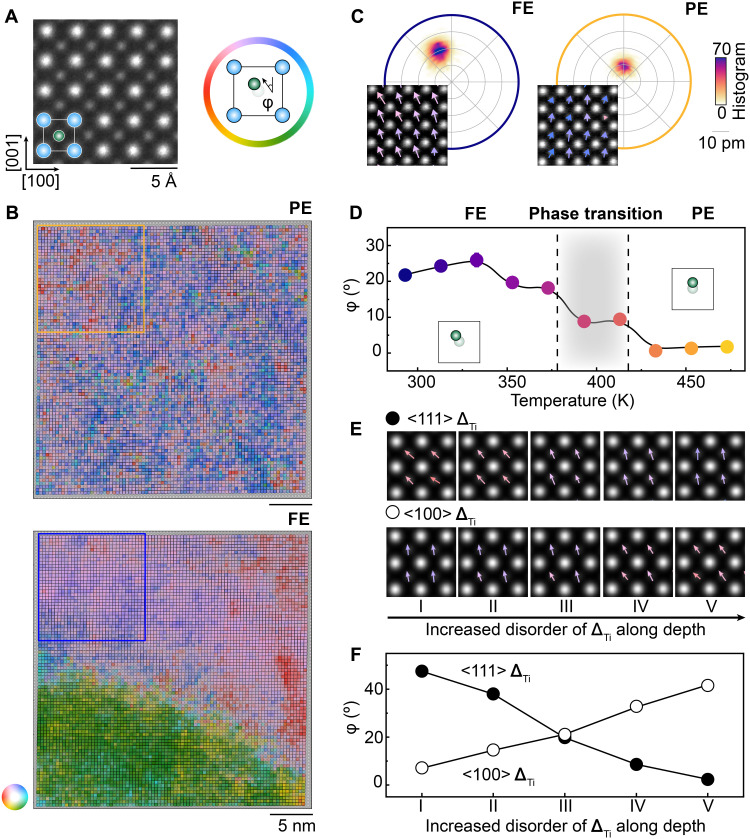
Finite Δ_Ti_ in the PE phase and evidence of <111> displacements. (**A**) Left: Zoomed-in ADF-STEM image of BaTiO_3_ overlapped with an atomic model showing Ba (blue) and Ti (green). The projection is along the [010] direction. Right: Definition of the direction of **Δ**_Ti_ (ϕ). (**B**) Real-space map of **Δ**_Ti_ in FE and PE phases. The color and transparent represent the direction and amplitude, respectively. (**C**) Polar histogram of **Δ**_Ti_ in FE and PE phases. The radius of the polar histogram plot is 40 pm. The inset shows the zoomed-in ADF-STEM image overlapped with measured **Δ**_Ti_ in the FE and PE phase. (**D**) Evolution of ϕ with temperature. The inset shows the schematic graph of the changes between FE and PE phases. The black dashed line and gray shadow highlight the phase transition region. The plot summarizes data from the region marked by a rectangle in (B) to exclude the effect of FE domain boundary. (**E**) Multislice image simulations and mapping of projected **Δ**_Ti_ with increasing disorder (I to V) along the depth (electron beam imaging direction). The top and bottom panels represent **Δ**_Ti_ along the <111> and <100> direction, respectively. (**F**) ϕ of the projected displacements from multislice simulations.

To understand the microscopic structure of this disorder, we quantify **Δ**_Ti_ in real space, which can be mapped quantitatively for each unit cell through fitting atomic positions in ADF-STEM images (see Supplementary Text). The top panel of Fig. 2A shows an ADF-STEM image of BaTiO_3_ at 473 K, where the Ba atomic columns appear brighter than Ti columns due to their larger atomic number. Figure 2B presents a large field-of-view ADF-STEM image overlaid with **Δ**_Ti_ at 473 K (PE) and 293 K (FE). The color represents the direction of **Δ**_Ti_ and transparency reflects the amplitude of the displacement. Even above *T_c_*, the PE phase exhibits short-range polar clusters with finite **Δ**_Ti_, close to 9 pm. Displacement magnitudes in the PE phase are larger than the 4-pm precision of the measurement (fig. S4). The ADF-STEM image reveals no apparent crystalline defects or boundaries that would pin polar displacements in the PE phase. The possible influence of oxygen vacancies generated at high temperatures is also excluded (fig. S5). Thus, these visualizations provide direct, real-space evidence for finite **Δ**_Ti_ well into the PE phase.

### <111> displacement direction

We next address the direction of **Δ**_Ti_, whether it inherits <001> tetragonal displacements or <111> rhombohedral displacements. To do so, we directly quantify the projected shift direction (ϕ), as defined in the right panel of Fig. 2A. In the FE phase, long-range order starts to emerge when compared with the PE phase (bottom panel of Fig. 2B), but the ϕ does not align well with either the <001> (ϕ = 0°) or <111> (ϕ = 45°) projected directions (left panel of Fig. 2C). Instead, ϕ in the FE phase aligns close to <001> projected direction (right panel of Fig. 2C). In addition, when we tracked the evolution of ϕ across the FE-PE phase transition, we find large changes within the phase transition temperature region (Fig. 2D; raw data are shown in fig. S6). This evolution is also reproducible across regions with different domain configurations (figs. S7 and S8), excluding the influence of domain boundaries.

We show that this abnormal alignment and change of ϕ represents the distinct disorder configurations <111> rhombohedral displacements in both the FE and PE phases. ADF-STEM is a projection imaging method along a column of atoms. To simplify, we only consider the disorder in the displacements along the depth direction instead of three-dimensional disorder to interpret changes in the projected displacements (see Supplementary Text). Multislice image simulations can be used to study the effect of disorder in projection. We construct supercells with random stackings of <111> and <001> **Δ**_Ti_ variants along the depth direction (figs. S8 and S9). Figure 2 (E and F) summarize the dependence of ϕ on the disorder of **Δ**_Ti_ along the depth direction. The ϕ determined in the experiment is reproducible in the case of rhombohedral-like <111> displacements, which shows a monotonic decrease from 45° to 0° when we increase disorder. In contrast, the tetragonal-like <100> displacement direction shows the opposite trend and is inconsistent with the experimental data in Fig. 2D. Therefore, the FE phase, nominally in the “tetragonal” phase below *T_c_*, comprises rhombohedral displacements, which, in projection STEM, manifest as ϕ that increases as temperature/disorder decreases. The temperature trend (Fig. 2D) and simulations of disorder (Fig. 2F) further indicate that the PE phase inherits <111> displacements but with increased disorder.

### Emergence of **Δ**_Ti_ correlations in real space

**Fig. 3.**
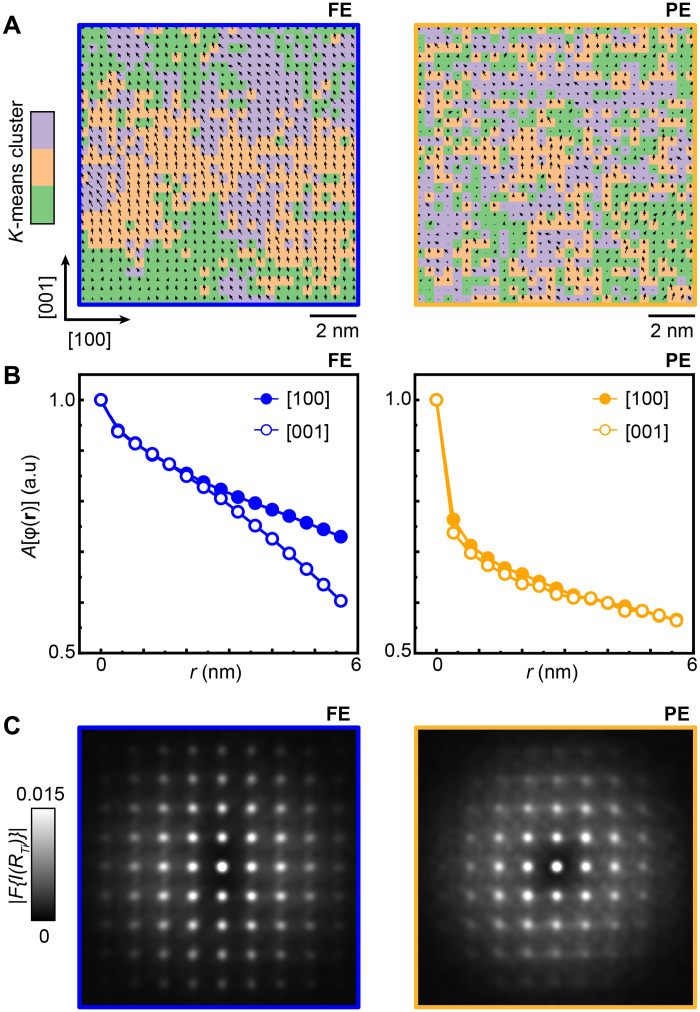
Real-space correlations of polar displacements Δ_Ti_. (**A**) *K*-means clustering of the region marked by rectangle in Fig. 2B; see details in Supplementary Text. The color represents individual clusters of correlated displacements. The arrows represent **Δ**_Ti_, with the size and length encoding the amplitude and direction, respectively. (**B**) Autocorrelation function of **Δ**_Ti_ {*A*[ϕ(**r**)]} extracted along [100] (solid circle) and [001] (hollow circle) directions. a.u., arbitrary units. (**C**) Fourier transform of 2D Ti positions in FE (left) and PE (right). Anisotropy in the diffuse intensity is evident.

To elucidate local correlations between nearby Ti sites and their relationship to diffuse intensity in diffraction, we visualize fluctuations in **Δ**_Ti_. Clustering analysis was performed to enhance the visibility of these fluctuations (see Supplementary Text for details). Clustering identifies nanoscale regions (colors) with different displacement directions/amplitudes (arrows). Despite having large FE domains, the FE phase still shows local fluctuations within a single domain, as captured by the clusters (Fig. 3A). In the PE phase, the clusters are smaller and more randomly distributed.

To quantify the real-space correlations, we calculated the two-dimensional (2D) autocorrelation function of **Δ**_Ti_ {*A*[ϕ(**r**)]} and extracted its decay behavior along two orthogonal directions, [100] and [001] (see Supplementary Text for more details). As shown in Fig. 3B, the decay of *A*[ϕ(**r**)] encodes the degree of correlations between **Δ**_Ti_, with a faster decay indicating weaker correlations. In the FE phase, an anisotropic correlation is evident, with slower decay along [100] indicating stronger correlation compared to [001]. In the PE phase, *A*[ϕ(**r**)] decays more rapidly and appears more isotropic.

The effect of local **Δ**_Ti_ correlations can further be linked to diffuse intensity in reciprocal space, through Fourier transformation of the 2D Ti sublattice (see Supplementary Text and figs. S10 and S11 for details). As summarized in Fig. 3C, the Fourier transform of Ti positions in the FE phase exhibits diffuse intensity along the [001] direction, consistent with anisotropic correlations revealed by *A*[ϕ(**r**)]. In the PE phase, however, diffuse intensity is observed along both orthogonal directions, which indicates a transition to isotropic behavior, also in agreement with *A*[ϕ(**r**)].

**Fig. 4.**
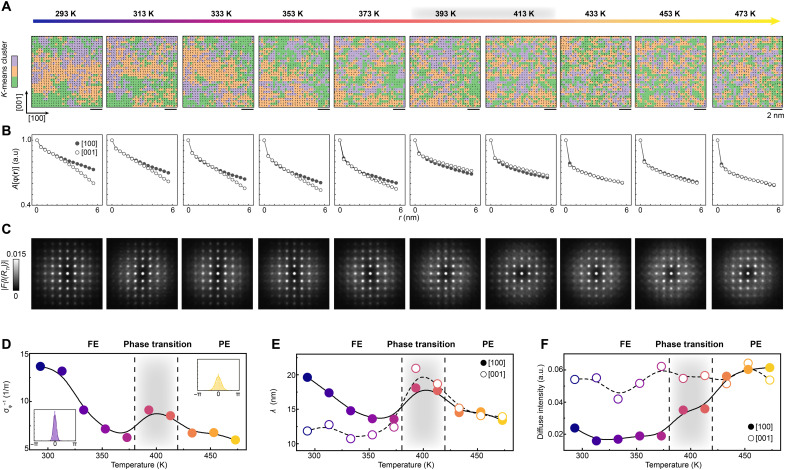
Evolution of real-space correlations of Δ_Ti_ across temperatures. (**A**) *K*-means clusters, (**B**) autocorrelation function of **Δ**_Ti_, and (**C**) Fourier transform result of 2D Ti positions measured across temperatures. (**D**) SD of the displacement direction, $\sigma_{\phi }^{-1}$, at different temperatures. The inset shows the histogram at 293 and 473 K, respectively. The mean value was set to 0. (**E**) Correlation length, (λ), extracted from *A*[ϕ(**r**)] at different temperatures. The hollow and solid circle represents the (λ) along the [001] and [100] direction, respectively. (**F**) Temperature-dependent diffuse intensity in the Fourier transform. The diffuse intensity is normalized to the (200) and (002) Bragg peaks. The hollow and solid circle represents the [001] and [100] direction, respectively. The black dashed line and gray shadow highlights the phase transition region.

Detailed in situ heating in the same field of view helps track the evolution of these parameters. The temperature-dependent behaviors of the fluctuations, correlation function, and diffuse intensity anisotropy are summarized in Fig. 4. Fluctuations become more notable across the phase transition (Fig. 4A), further quantified by the SD of ϕ. As exhibited in Fig. 4D, σ_ϕ_ grows with increasing temperature in both FE and PE phases (raw data are shown in fig. S14). Figure 4B shows the *A*[ϕ(**r**)] along [100] and [001] direction at different temperatures, confirming that anisotropy vanishes as the system transits through the phase transition region. Quantification of correlation lengths (λ; see Supplementary Text for more details) along two directions (Fig. 4E) shows the same trend. The difference of λ between two orthogonal directions begins to disappear when BaTiO_3_ crosses the FE-PE transition. Likewise, diffuse intensity in Fourier transform follows the same trend. As displayed in Fig. 4 (C and F), diffuse intensity becomes isotropic across the phase transition region, as revealed through linecuts along the [001] and [100] directions (fig. S15). The evolution of diffuse intensity in Fourier transform is also consistent with that observed in diffraction pattern (fig. S16). These three parameters show a high degree of consistency, demonstrating that local correlations of **Δ**_Ti_ dictate the FE-PE phase transition.

### Capturing all phase transitions in BaTiO_3_ under the O-D framework

**Fig. 5.**
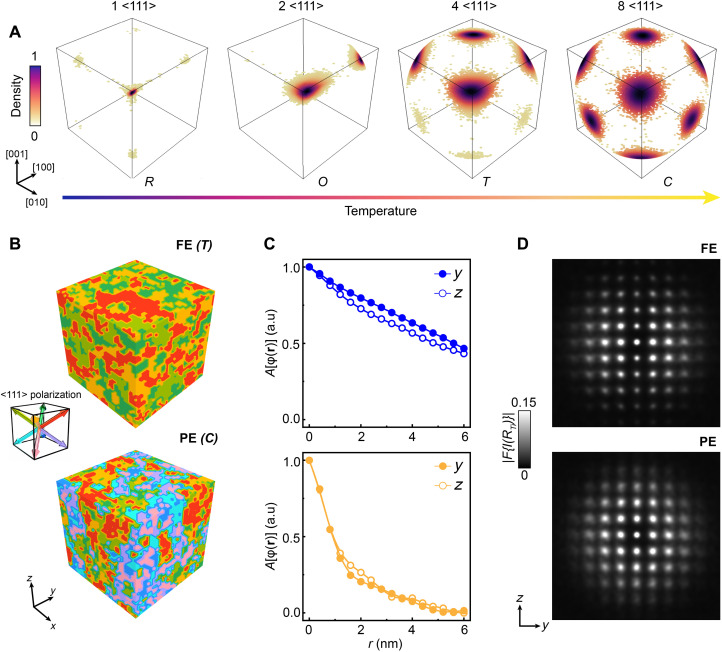
Phase-field simulations of an O-D mechanism in BaTiO_3_. (**A**) Occupation of Ti displacement direction position at different temperatures collected from phase-field simulations. The density is normalized from 0 to 1 for each temperature. (**B**) Distribution of eight <111> **Δ**_Ti_ within a 32 by 32 by 32 supercell in the FE and PE phase. (**C**) Autocorrelation function of *yz*-plane projected Ti displacements (*A*[ϕ(**r**)]). The solid and hollow circle represents the profile extracted along the *y* (solid circle) and *x* (hollow circle) direction. (**D**) Fourier transform of 2D image reconstructed from Ti displacements projected on the *y*-*z* plane. The raw data of projected Ti displacements and reconstructed 2D patterns are shown in fig. S17.

The atomistic mechanism of the O-D model we identified in BaTiO_3_, involving (i) persistently finite **Δ**_Ti_ in the PE phase, (ii) displacements along the <111> directions, and (iii) the key role of correlations of **Δ**_Ti_, motivates a simple phase-field simulation to replicate the phase transitions in BaTiO_3_. Starting from our experimental evidence, we only considered <111>-type **Δ**_Ti_ and incorporated the interplay between correlation of **Δ**_Ti_ and thermal fluctuations in a phase-field simulation (see Materials and Methods). As displayed in Fig. 5A, the occupation probabilities of <111> directions undergo three distinct changes, mirroring the typical phase transition sequence in BaTiO_3_ (R-O-T-C), consistent with other theoretical works (*44*–*46*).

We further mapped the spatial distribution of <111> **Δ**_Ti_ within a 32 by 32 by 32 supercell for both FE and PE phases and projected the **Δ**_Ti_ onto the *yz* plane (fig. S17). As depicted in Fig. 5B, the fluctuations of **Δ**_Ti_ are evident in both the FE and PE phases, with more evident fluctuations observed in the PE phase. To quantify the correlations, we extracted *A*[ϕ(**r**)] along *y* and *z* directions and performed Fourier transform of positional pattern, analogous to the STEM analysis. Figures 5C shows the *A*[ϕ(**r**)] extracted along two directions in FE and PE phases, with decays that align with the trends found in experimental data. There is an anisotropic decay along two directions in the FE phase, whereas the PE phase exhibits isotropic and faster decay. Furthermore, the Fourier transform shown in Fig. 5D reveals changes in diffuse intensity that mirror those in the experiment.

## DISCUSSION

In this work, we present direct real-space visualizations of FE order parameters in BaTiO_3_ across the FE-PE phase transition. With the help of real-space visualization, we provide atomistic insights into the controversies of the O-D model in BaTiO_3_. (i) Finite **Δ**_Ti_ are evident in the PE phase. (ii) The displacement direction of **Δ**_Ti_ in the PE phase, inherited from the FE phase, remains aligned along <111> directions. (iii) **Δ**_Ti_ show distinct local correlations across temperatures, and their variation between anisotropy and isotropy generates diffuse intensity in reciprocal space. Last, all these parameters undergo substantial changes across the phase transition region. This work demonstrates the O-D nature of phase transition in BaTiO_3_ and highlights the critical role of atomic-scale visualizations in revealing local correlation within the ordered and disordered states. Furthermore, our phase-field model will help determine the role of O-D transitions in mesoscale domains and FE-related properties of BaTiO_3_ and other perovskite ferroelectrics.

## MATERIALS AND METHODS

### Sample preparation

BaTiO_3_ is a commercial single crystal (MTI Corporation). The cross-sectional STEM specimens were prepared using standard gallium focused ion beam (Thermo Fisher Scientific Helios) lift-out and thinning. The samples were thinned down using an accelerating ion voltage of 30 kV with a decreasing current from 100 to 40 pA and then with a fine polishing process using an accelerating voltage of 5 and 2 kV and a current of 41 and 23 pA. The thickness of our BaTiO_3_ lamella was estimated to be ~30 nm by zero-loss peak in electron energy-loss spectroscopy (*47*).

### In situ XRD measurement

Single-crystal BaTiO_3_ was first fixed to the heater holder with colloidal silver to ensure good thermal contact. The θ-2θ scanning was carried out with a high-resolution diffractometer (Smartlab, Rigaku) using monochromatic Cu *K*_α1_ radiation (λ = 1.5406 Å) in an atmosphere of argon gas at different temperature stages. The temperature range was from 20° to 200°C with a 20°C step. All temperature-dependent measurements were taken 5 min after the temperature had reached the set point. The lattice parameters of a- and c-domain were calculated from the position of the diffraction peak (002), which were determined via piecewise Gaussian fitting of both separate peaks.

### Scanning transmission electron microscopy

In situ STEM experiments were performed in an aberration-corrected microscope (Thermo Fisher Scientific Themis Z G3) operated at 200 kV. The in situ heating experiment was carried out using micro-electromechanical systems (MEMS)-based heating/bias chips (DENSsolutions). The temperature range was from 20° to 200°C with a 20°C step. ADF-STEM images at all temperatures were collected using an 18.9-mrad convergence angle and 30-pA probe current. The collection angles ranged from 68 to 200 mrad, corresponding to high-angle ADF. This imaging mode is mostly sensitive to atomic number and has interpretable contrast, making quantification of atomic positions reliable. To minimize image drift and obtain a high signal-to-noise ratio, fast-acquisition frames were collected. Each frame was acquired with 2048 × 2048 pixels and a 100-ns dwell time. Fifty frames in total were collected and aligned using a rigid registration method optimized for noisy image frames (*48*).

### Phase-field simulation

In traditional FE phase-field models, a position-dependent spontaneous polarization **P** and a displacement field ***u*** serve as order parameters. Polarization dynamics is governed by the time-dependent Ginzburg-Landau equation, whereas the corresponding equilibrium equations capture the equilibrium of stress and electric fields (*49*)

$$\frac{\partial P}{\partial t}=-L\frac{\mathrm{\delta F}}{\delta P},\;\nabla \cdot \text{sigma}=0,\;\nabla \cdot D=\rho_{f}$$ (1)

where *L* is a kinetic coefficient related to domain wall mobility, *F* is the total free energy of the system, $\frac{\mathrm{\delta F}}{\delta P}$ is the thermodynamic driving force for polarization evolution, $\sigma_{\mathrm{ij}}$ is the stress tensor, D is the electric displacement, $\rho_{f}$ is the free charge density, and ***r*** and *t* are the spatial coordinate and time, respectively. The thermal field $E^{\text{thermal}}$ follows (*50*)

$$\begin{array}{c} \langle E_{i}^{\text{thermal}}\left(x_{1},t_{1}\right)E_{j}^{\text{thermal}}\left(x_{2},t_{2}\right)\rangle =\\ 2k_{B}\mathrm{TL\delta}\left(x_{1}-x_{2}\right)\delta \left(t_{1}-t_{2}\right) \end{array}$$ (2)

where $k_{B}$ is the Boltzmann constant and *T* is the thermodynamic temperature. The total free energy of a bulk system can be defined as follows

$$F=\int_{V}\left(f_{\text{Land}}+f_{\text{grad}}+f_{\text{elastic}}+f_{\text{elec}}\right)\;\mathrm{dV}$$ (3)

where *F* includes the bulk free energy $f_{\text{Land}}\left(P\right)$, domain-wall energy $f_{\text{grad}}$, elastic energy $f_{\text{elastic}}$, and electrostatic energy $f_{\text{elec}}$, with E as the applied static electric field. The corresponding energy densities are $f_{\text{Land}},f_{\text{grad}},f_{\text{elastic}}$, and $f_{\text{elec}}$.

The bulk free-energy density is expressed as a sixth-order polynomial expansion

$$\begin{array}{c} f_{\text{Land}}=a_{1}\left(P_{1}^{2}+P_{2}^{2}+P_{3}^{2}\right)+a_{11}\left(P_{1}^{4}+P_{2}^{4}+P_{3}^{4}\right)\\ +a_{12}\left(P_{1}^{2}P_{2}^{2}+P_{2}^{2}P_{3}^{2}+P_{1}^{2}P_{3}^{2}\right)\\ +a_{111}\left(P_{1}^{6}+P_{2}^{6}+P_{3}^{6}\right)\\ +a_{112}\left[P_{1}^{4}\left(P_{2}^{2}+P_{3}^{2}\right)+P_{2}^{4}\left(P_{1}^{2}+P_{3}^{2}\right)\right.\\ \left.+P_{3}^{4}\left(P_{1}^{2}+P_{2}^{2}\right)\right]+a_{123}P_{1}^{2}P_{2}^{2}P_{3}^{2} \end{array}$$ (4)

where *a*_1_ to *a*_123_ are Landau parameters.

The gradient energy density in an anisotropic system can be calculated by

$$f_{\text{grad}}=\frac{1}{2}g_{\text{ijkl}}P_{i,j}P_{k,l}$$ (5)

where $g_{\text{ijkl}}$ is the gradient energy coefficient and $P_{i,j}=\frac{\partial P_{i}}{\partial x_{j}}$. The elastic energy density is given by

$$f_{\text{elastic}}=s_{\mathrm{ij}}\sigma_{\mathrm{ij}}^{2}$$ (6)

where $s_{\mathrm{ij}}$ is the compliance coefficient. Here, $\sigma_{\mathrm{ij}}=C_{\text{ijkl}}\left(\varepsilon_{\mathrm{kl}}-\varepsilon_{\mathrm{kl}}^{0}\right)$, with $C_{\text{ijkl}}$ as the stiffness tensor, $\varepsilon_{\mathrm{ij}}$ as the strain tensor, and $\varepsilon_{\mathrm{ij}}^{0}$ as the eigenstrain, defined as

$$\varepsilon_{\mathrm{ij}}^{0}=Q_{\text{ijkl}}P_{k}P_{l}$$ (7)

where $Q_{\text{ijkl}}$ is the electrostrictive coefficient tensor.

The electrostatic energy density $f_{\text{elec}}$ in the phase-field simulation is given by

$$f_{\text{elec}}=-P_{i}\left(r\right)E_{i}\left(r\right)-\frac{1}{2}P_{i}\left(r\right)E_{i}^{\text{in}}\left(r\right)$$ (8)

where $E_{i}^{\text{in}}\left(r\right)$ is the *E*-field induced by the dipole moments, and $E_{i}\left(r\right)$ is the applied electric field.

To reproduce the phase transition from R phase to O, T, and C phases, we use $a_{1}=a_{0}\left(T-T_{c}\right)$, with other Landau parameters fitted to represent different FE polarization phases in specific directions. Our phase-field simulation is based on an eight-site model of BaTiO_3_. In our model, only the R phase potential well is introduced, with fixed Landau parameters as the temperature increases, showing rhombohedral-like local displacements at all temperatures. The phase transition originates from competition between thermal fluctuations and nearest-neighbor (including second and third nearest neighbor) interactions. As shown in ref. (*39*), interactions were added to penalize dipole misalignment in first, second, and third nearest-neighbor sites. In the mesoscale phase-field model, this is simplified to anisotropic fluctuations in different directions, with the thermal field given by

$$E_{i}^{\text{thermal}}\left(x,t\right)=\eta \left(x,t\right)\sqrt{\frac{2k_{B}\mathrm{T\gamma}}{\tau \Delta V}}-b_{i}$$ (9)

where η is a random vector, γ is the damping coefficients of polarization evolution, ΔV is the volume of a grid cell, and τ is the time step. $b_{i}$ is the reduced anisotropic temperature fluctuation field parameters. The term $b_{i}$ is then derived by reproducing the R-O-T-C transition within a reasonable transition temperature range.

The other parameters (all in SI units) are listed in Table 1.

**Table 1.** Parameters used in phase-field simulation.

*a*_1_	−3.8 × 10^8^ m^2^·N/C^2^
*a*_11_	4.8 × 10^8^ m^6^·N/C^4^
*a*_12_	1.9 × 10^8^ m^6^·N/C^4^
*Q*_11_	0.1 m^4^/C^2^
*Q*_12_	−0.034 m^4^/C^2^
*Q*_44_	0.029 m^4^/C^2^
*s*_11_	9.1 × 10^−12^ m^2^/N
*s*_12_	−3.2 × 10^−12^ m^2^/N
*s*_44_	8.2 × 10^−12^ m^2^/N
γ	0.05 J·m/(A·s^2^)
τ	5 × 10^−12^ s
*a*_0_	3.4 × 10^8^ J/m^3^
*p*_0_	0.5 C/m^2^
*b*_2_	0.8
*b*_3_	1.2
*g*_11_	0.6

The simulation scale is 32Δx×32Δy×32Δz, with grid scales Δx and Δz at 1 nm. Fourier methods were used to solve the equations. Open circuit electrical boundary conditions, and periodic mechanical boundary conditions were adopted in the calculations.

## Supplementary Materials

### This PDF file includes:

Supplementary Text Figs. S1 to S18 References
