# Supplementary material for: Real-space visualization of order-disorder transition in BaTiO3
Source: Sci Adv. 2025 Sep 3;11(36):eadx9804. doi: 10.1126/sciadv.adx9804 (PMC12407081; doi:10.1126/sciadv.adx9804)
Supplement: Supplementary file 1 — Supplementary Text Figs. S1 to S18 References [file sciadv.adx9804_sm.pdf]

Science Advances
AAAS

Supplementary Materials for

Real-space visualization of order-disorder transition in $BaTiO_3$

Yang Zhang *et al.*

Corresponding author: Yang Zhang, yzhang6@fas.harvard.edu; Ismail El Baggari, ielbaggari@fas.harvard.edu

Sci. Adv. **11**, eadx9804 (2025)
DOI: 10.1126/sciadv.adx9804

This PDF file includes:

Supplementary Text
Figs. S1 to S18
References

Supplementary Text

I. Diffuse intensity in reciprocal space

In reciprocal space, the positional disorder and chemical disorder have different effects on the diffuse intensity.

Displacement (Δ) in a lattice appears in reciprocal space as:

$$F(\mathbf{k}) = \sum_j f_j \exp[i\mathbf{k} \cdot (\mathbf{r}_j + \Delta)] \tag{S1}$$

Typically, Δ is small, so we can perform a Taylor expansion of the exponential:

$$F(\mathbf{k}) = \sum_j f_j \exp(i\mathbf{k} \cdot \mathbf{r}_j) \left[1 + i(\mathbf{k} \cdot \Delta) - \frac{1}{2}(\mathbf{k} \cdot \Delta)^2 + \cdots\right] \tag{S2}$$

The Fourier amplitude thus scales as $|\mathbf{k} \cdot \Delta|^2$, which leads to diffuse intensity suppression at: (a) low $|\mathbf{k}|$ and when (b) $\mathbf{k}$ and Δ are orthogonal, as shown in Fig. S3B.

In contrast, chemical disorder appears in reciprocal space as:

$$F(\mathbf{k}) = \sum_j (f_j + \Delta f) \exp(i\mathbf{k} \cdot \mathbf{r}_j) \tag{S3}$$

This means the diffuse intensity in reciprocal space follows the same dependence as Bragg peaks, decaying monotonically with increasing $|\mathbf{k}|$, as shown in Fig. S3C.

II: Mapping Ti off-center shift (ΔTi)

To quantify the ΔTi, we first determined the position of the atomic columns by fitting 2D Gaussian functions to ADF-STEM images using both custom-built code and the open-source software Atomap (*51*). ΔTi was then measured based on the offset of B-site atomic columns relative to the reference high-symmetry position, or the center of mass determined from neighboring A-site ions (*52*). This measurement does underestimate the polar displacement in $BaTiO_3$, as the order parameter originates from the off-center shift of Ti relative to its six neighboring O atoms. Nevertheless, it is a more robust and established method for determining tiny atomic shifts in STEM measurements (*53–55*).

The maximum amplitude of ΔTi was normalized to 1 for all images shown in the main text and Supplementary Information. The real amplitude values were shown in the polar histogram figures.

III: Disorder along the beam direction

Since the ADF-STEM image is a projected image, disorder along the beam direction will affect the projected ΔTi. At lower temperatures, long-range order begins to emerge as the material enters the ferroelectric (FE) phase. Therefore, disorder along the beam direction is expected to decrease compared to the paraelectric (PE) phase.

To examine the effect of disorder along the beam direction on the projected ΔTi, we employed multislice simulations with varying disordered configurations along the beam path. The simulations were carried out using `abTEM` (*56*) and custom Python scripts. Structural models with different correlation lengths along the beam direction were constructed. The simulation thickness was set to approximately 20 nm, with a slice thickness of 0.1 nm. The frozen phonon method was applied with 10 configurations per slice. Experimental parameters, including acceleration voltage, convergence angle, and collection angle, were used, with defocus set to 0 nm. Poisson noise was added to the simulated images.

As shown in Figs. S9–S10, increasing the disorder of $\langle 111 \rangle$-like displacements along the beam direction causes the projected ΔTi to align along the $\langle 001 \rangle$ direction, consistent with what we observe in the PE phase. In contrast, reduced disorder along the beam direction causes the projected ΔTi to deviate toward the $\langle 100 \rangle$ direction.

The variation in $\langle 111 \rangle$-like disorder along the beam direction closely resembles the trend observed in our experiments (Fig. 2), whereas the $\langle 100 \rangle$-like ΔTi produces an opposite behavior.

In the multislice simulations, we considered only the disorder along the depth (z) direction to isolate the effects on the projected ΔTi for simplification. In contrast, our phase-field simulations incorporate full three-dimensional disorder and yield the same trend for projected ΔTi (Fig. S18).

IV. Isolating positional disorder with the Fourier transformation.

To isolate the positional disorder in the Fourier transform, we first collected the fitted positions of the Ti sites in the ADF-STEM image ($\mathbf{R}_{\mathrm{Ti}}$). Then, we reconstructed a 2D pattern based on these fitted positions, denoted as $I(\mathbf{R}_{\mathrm{Ti}})$, ensuring that each 2D Gaussian peak had the same amplitude and radius. Finally, we performed a Fourier transform on the reconstructed 2D pattern, i.e., $\mathcal{F}\{I(\mathbf{R}_{\mathrm{Ti}})\}$. A Hann window was applied to reduce spectral leakage during the Fourier transform.

In this way, we could eliminate other contributions to the diffuse intensity and ensure that the resulting intensity originates solely from positional disorder. Figure S11 summarizes the analysis workflow. The FFT result shown in Fig. 3C is based on the reconstructed image presented in Fig. S12.

V. K-means clustering analysis.

To identify the correlated patterns in real space, we applied K-means clustering to the d_x and d_y components of the ΔTi. A 30×30 grid within the domain was selected to exclude the influence of the domain boundaries.

The number of clusters was set to $n = 3$, determined using the Elbow Method and Silhouette Score to ensure a balance between compactness and separation of clusters. We also tested different cluster numbers, which yielded consistent results (Fig. S13). To ensure reproducibility, the random initialization of centroids was controlled by using a fixed random seed.

VI. Auto-correlation function.

In our analysis, our goal is to quantify the degree of order in the Ti polar displacement (ΔTi), we thus construct an order parameter $|\Delta \mathrm{Ti}| \cdot \exp(i\varphi)$, with $|\Delta \mathrm{Ti}|$ as the amplitude and φ as the direction of the polar displacement. In this way, the 2D autocorrelation function of $|\Delta \mathrm{Ti}| \cdot \exp(i\varphi)$ matrix decay monotonically with increasing distance. Then we extracted the real part of the auto-correlation function result, and profiles along the [100]- direction and [010] direction are extracted for comparison.

To quantify the decay behavior of autocorrelation function, we calculate the correlation length (λ), which help determine characteristic decay length. Since the autocorrelation function follows exponential decay, we can fit it using the standard form

$$y = A \cdot \exp\left(-\frac{r}{\lambda}\right). \tag{S4}$$

To obtain λ, we converted autocorrelation function to logscale and then performed linear fitting using the transformed expression to get λ.

$$log(y) = \log(A) - \frac{r}{\lambda} \tag{S5}$$

VII. Standard deviation of ΔTi **direction**

To quantify the distribution of ΔTi direction, we calculate the standard deviation σ_φ. The normal distribution was carried out to fit the data and mean value was set as 0 (Fig. S13). The half width at half maximum (HWHM) was measured and the standard deviation was estimated using the relation:

$$sigma_\varphi = \frac{\text{HWHM}}{\sqrt{2\ln 2}} \tag{S6}$$

Finally, the inverse of the standard deviation, σ_φ^{-1} was shown in Fig. 4D.

VIII. Quantification of diffuse intensity in Fourier transform.

To quantify the evolution of diffuse intensity in the Fourier transform, we first extracted the line profiles along the [001] and [100] directions (dashed lines in Fig. S15). The line profile is then normalized to the the (200) and (002) Bragg peaks. The diffuse intensity is calculated by the integration between Bragg peaks.

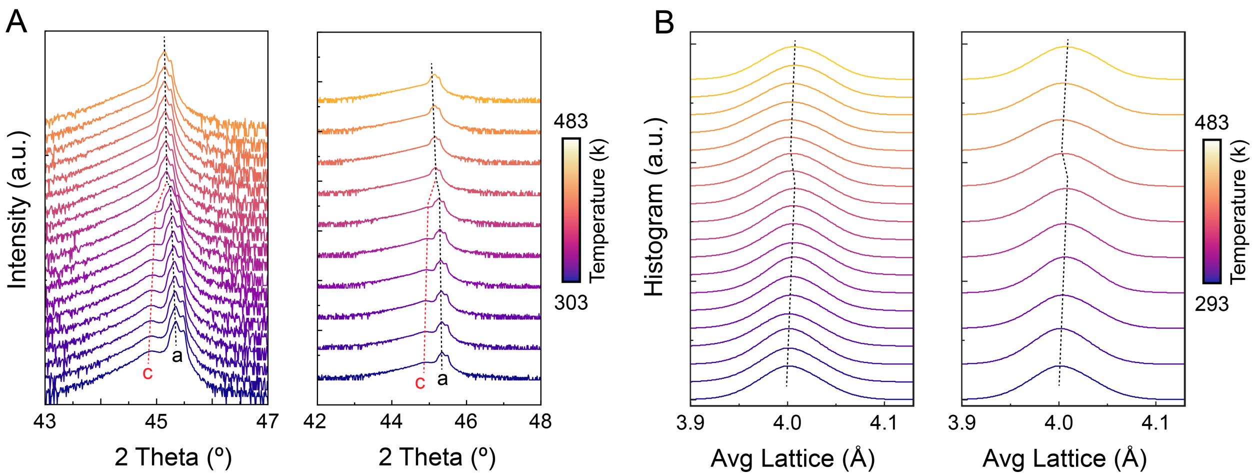

Figure S1: **Raw data of determining changes of lattice constant with temperatures.** (A) XRD data presented with 10 K and 20 K intervals. (B) Lattice constant measured from ADF-STEM image presented with 10 K and 20 K intervals. The curve is the normal fitting of the histogram.

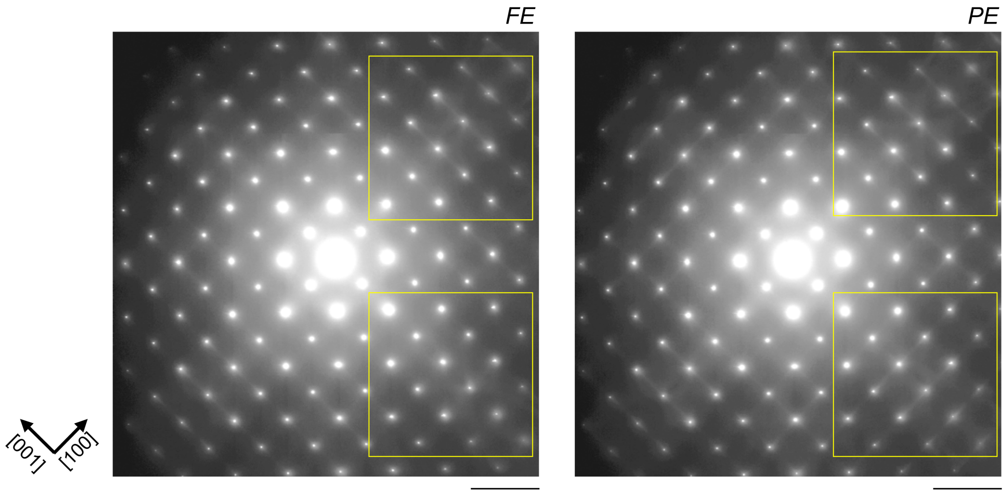

Figure S2: **Raw data of diffraction pattern collected from FE and PE phase.** The yellow rectangles show the cropped region shown in Fig. 1. The scale bar is 5 1/nm.

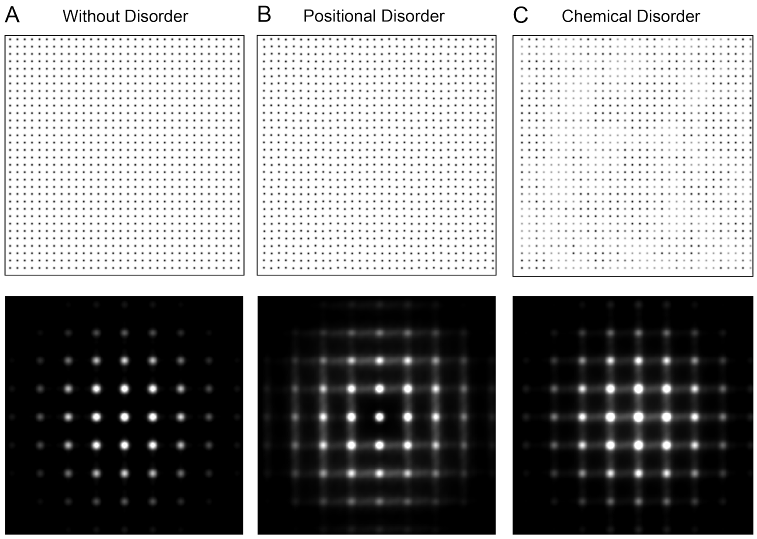

Figure S3: **Simulated reciprocal-space behavior of positional disorder and chemical disorder.** For positional disorder, the diffuse intensity is notable at high k-index spot (B), whereas the diffuse intensity is centered around center spot for chemical disorder (C).

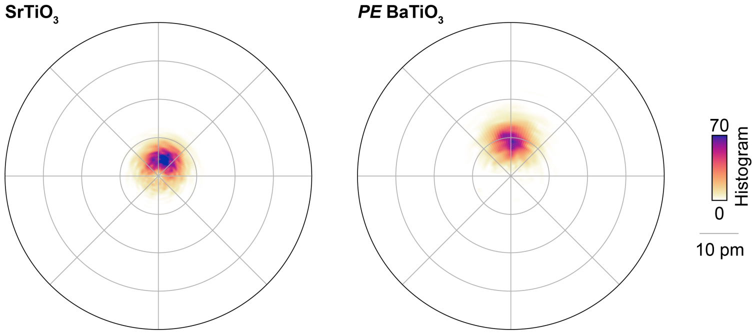

Figure S4: **Comparison of** ΔTi **between in** $\mathbf{SrTiO_3}$ **and paraelectric** $\mathbf{BaTiO_3}$**.** The ΔTi in $SrTiO_3$ can be estimated as measured precision ($\tilde{4}$ pm), which is lower than that of paraelectric $BaTiO_3$ ($\tilde{9}$ pm). Note that $SrTiO_3$ has small intrinsic displacements [9] so the precision of the measurement is expected to be even better than 4 pm.

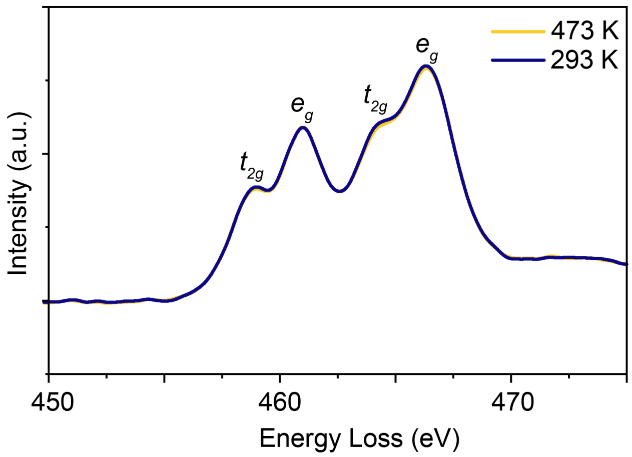

Figure S5: **Possible influence of oxyen vacancy.** Ti *L* signal measured at 300 K and 473 K. No evident difference between two signals means that no large amount of oxygen vacancies is formed at high temperature.

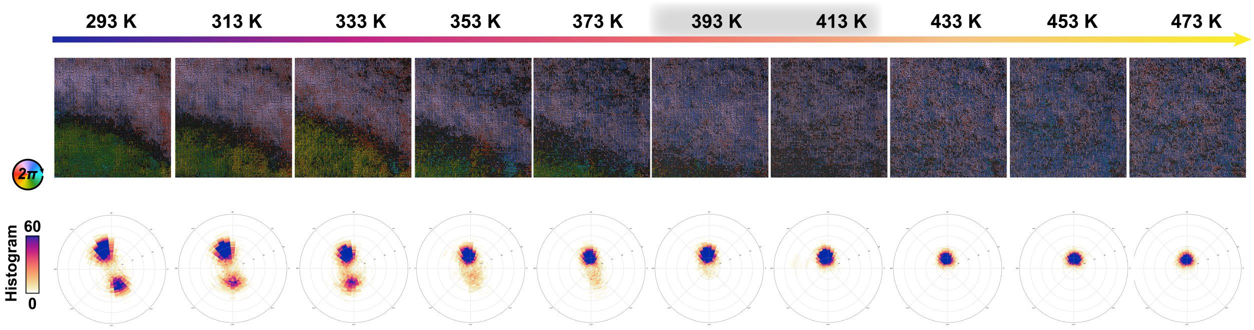

Figure S6: **Raw data of measured** ΔTi **at different temperatures.** Evolution of real-space distribution of ΔTi (upper panel) and polar histogram (lower panel) with temperatures.

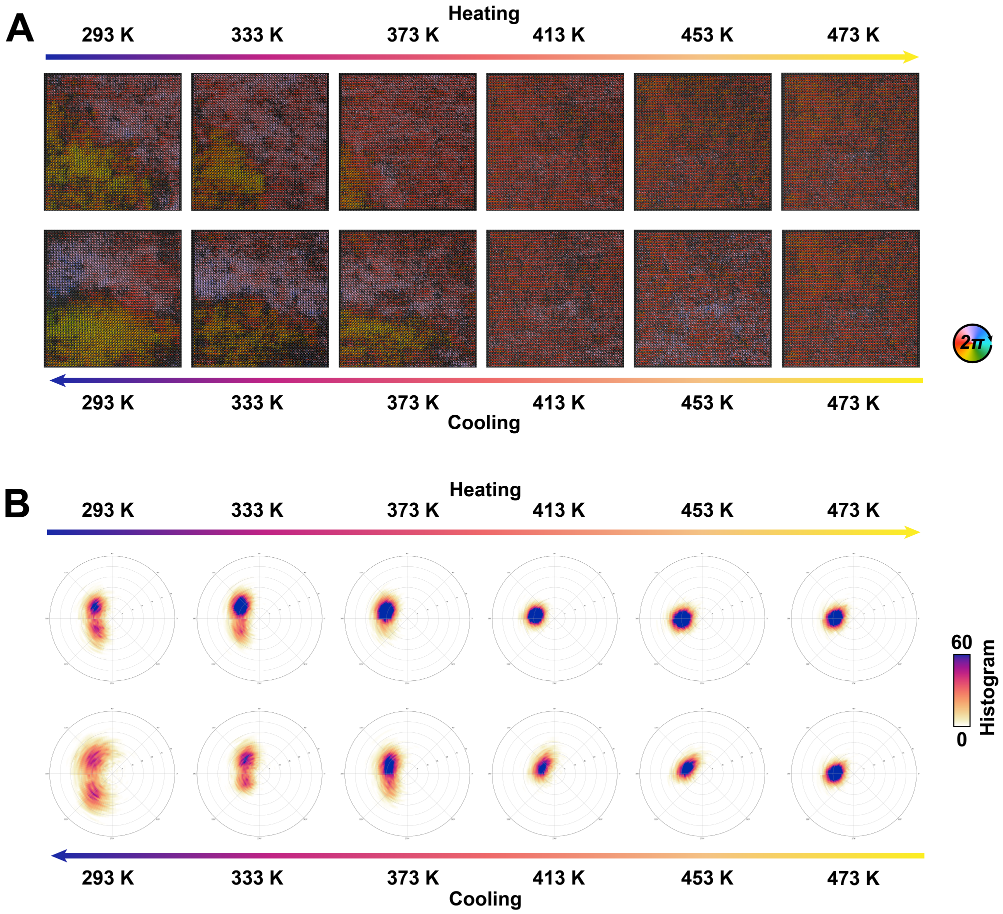

Figure S7: **Another dataset of measured** ΔTi **at different temperatures.** Real-space distribution of ΔTi (A) and polar histograms (B) at selective temperatures. Similar evolution with 180 ° domain is determined for 90○ domain configuration.

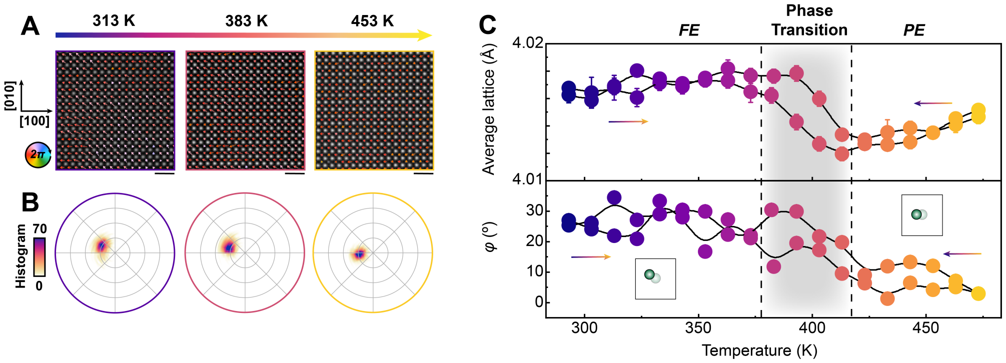

Figure S8: **Analysis of the** ΔTi **for a 90 ° domain configurations.** (A) The zoom-in ADF-STEM image overlaid with measured ΔTi and (B) polar histogram of ΔTi acquired from a single domain shown in Fig. S6. The radius is 0 to 40 pm. Three temperatures were selected. (C) Evolution of lattice constant (upper panel) and Ti shift direction (ϕ, lower panel) with temperatures. The data during heating and cooling cycle is collected. The arrow represents the heating and cooling curve. The inset shows the schematic graph of ΔTi in FE and PE phase. The black dashed line and gray shadow determines the phase transition region.

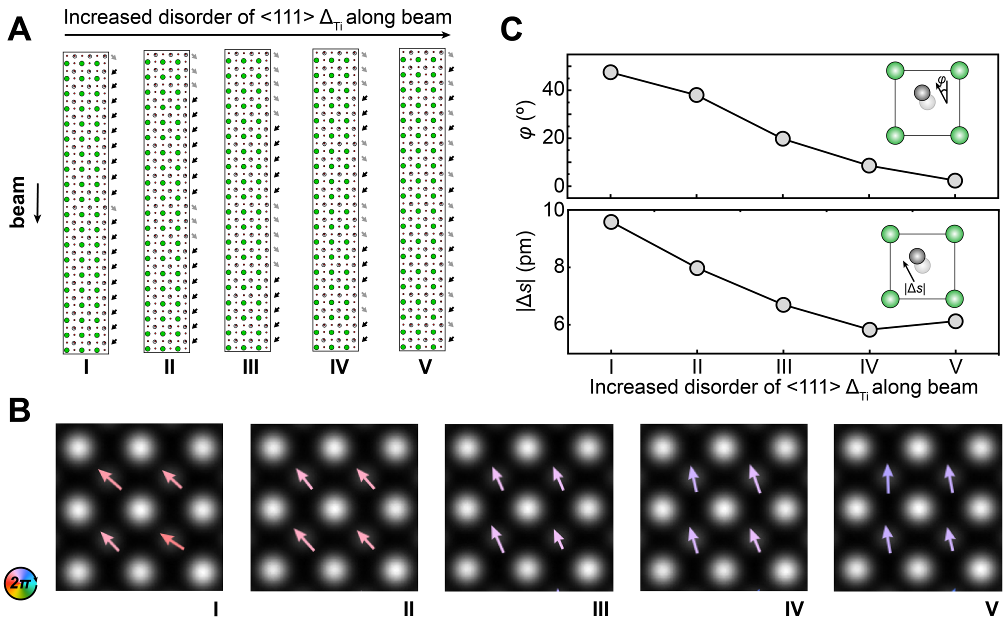

Figure S9: **Simulation of projected** ΔTi **with different degrees if disorder is in** <**111**>**-like displacements along the beam direction.** (A) Five types of supercells with increasingly more disordered configurations along the beam direction. Correlation between Ti displacements is gradually suppressed from I to V. The arrow shows the Ti shift direction along one of the eight <111> displacement directions. (B) Multislice simulation of ADF-STEM images of the five super cells. The arrow shows the measured Ti shift direction. (C) Ti shift direction (ϕ, upper panel) and amplitude ($\Delta\mathbf{s}$, lower panel) measured from simulated images.

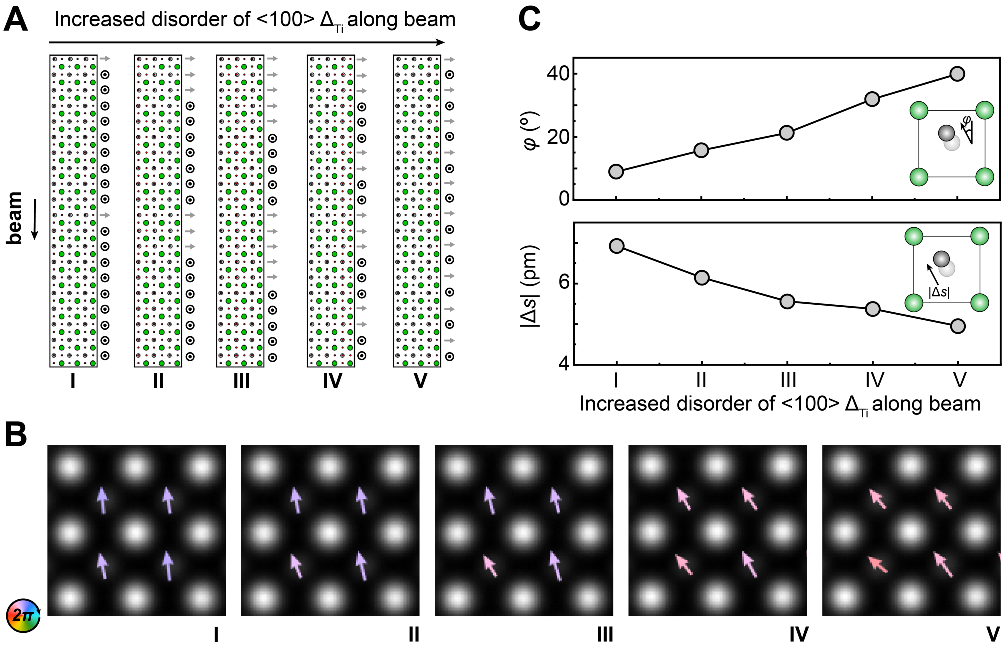

Figure S10: **Simulation of projected** ΔTi **with different degrees if disorder is in <100>-like displacements along the beam direction.** Same analysis with Fig. S9, but now considering ΔTi shift directions along the tetragonal <100>-like directions.

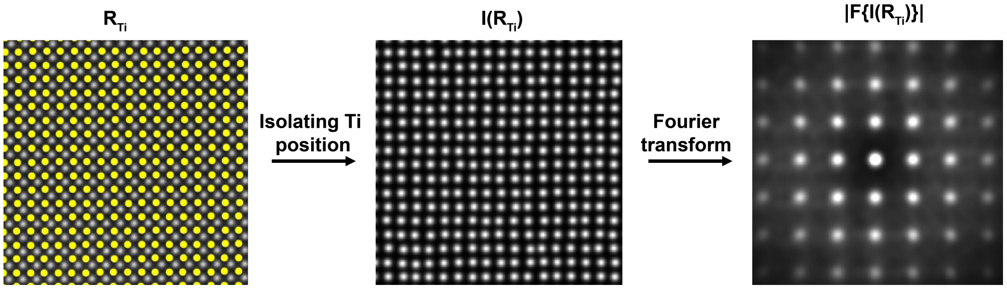

Figure S11: **Fourier transform of isolated Ti positions.** The left panel is the fitted 2D gaussian position of Ti (R_{Ti}). The middle panel is the reconstructed 2D gaussian pattern with fitted position ($I(R_{Ti})$). The right panel is the module of Fourier transform of $I(R_{Ti})$.

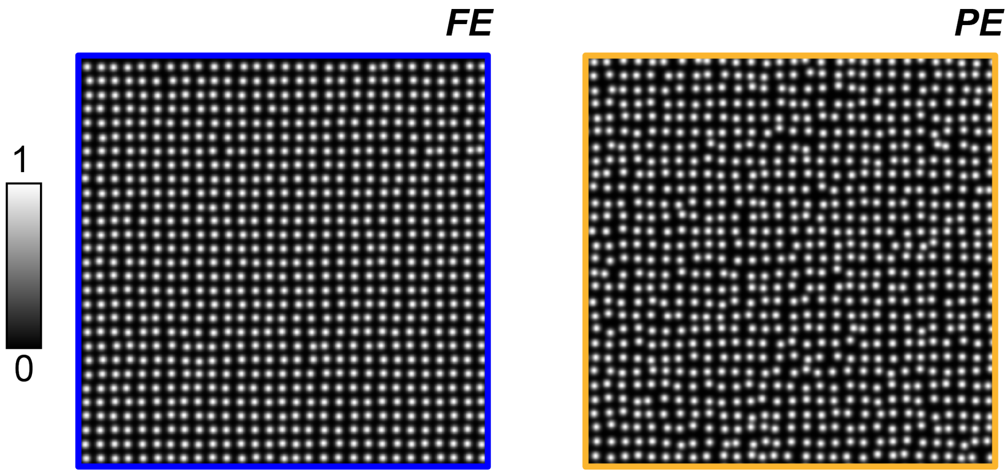

Figure S12: **Isolated Ti positions in FE and PE phases.** The displacement of Ti is magnified five times to show the difference between the two phases. Details about how to get the 2D pattern are shown in Materials and Methods.

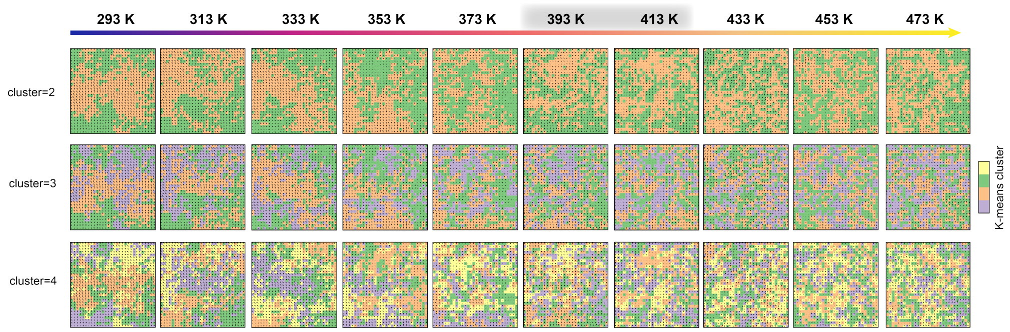

Figure S13: **Evolution of K-means cluster with temperatures.** Different clusters (n=2, 3, and 4) were tested, all of which shows same tendency.

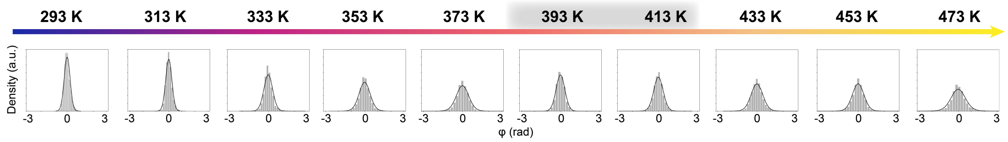

Figure S14: **Histogram of direction of ΔTi (ϕ) at different temperatures.** The mean value is centered at 0. The half width at half maximum (HWHM) is measured to calculate the standard deviation of ϕ

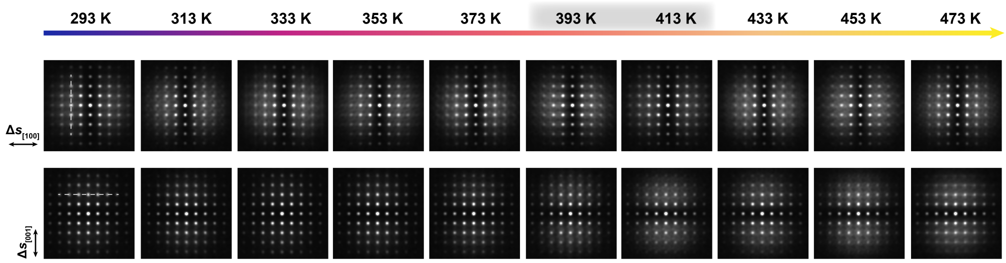

Figure S15: **Temperature dependence of diffuse intensity in the Fourier transform.** The upper and lower panel represent the diffuse intensity contributed by ΔTi component along [100] and [001] direction separately. The dashed line represents the position for linecuts.

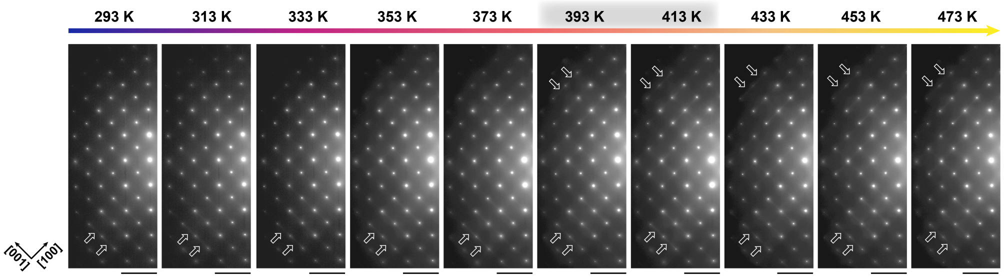

Figure S16: **Temperature-evolution of selected area electron diffractions.** The arrow highlights the diffuse lines at high index position.

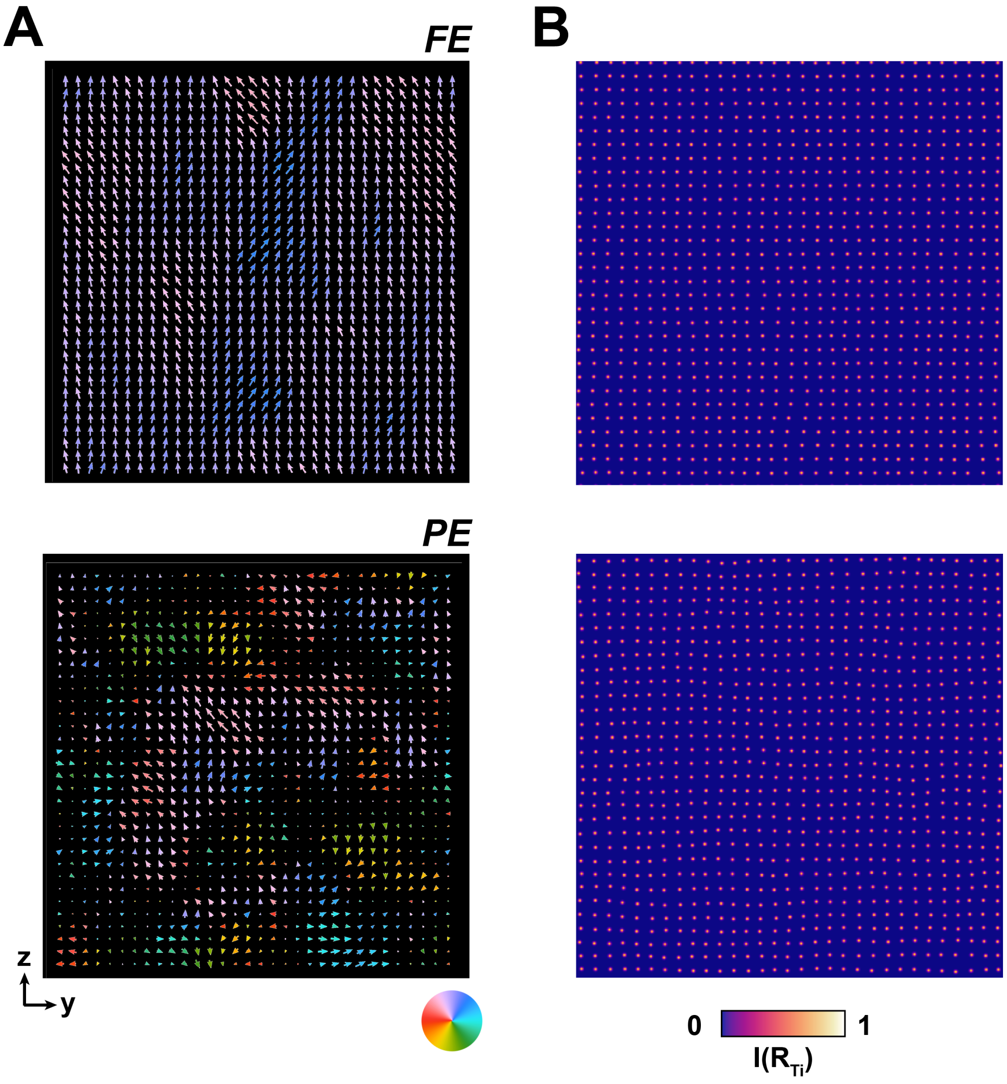

Figure S17: **Analysis of** ΔTi **in the phase field simulation.** (A) yz-plane projected ΔTi of FE and PE phase collected from phase-field simulation. (B) 2-D pattern reconstructed by ΔTi. This image is used for the FFT shown in Fig. 5.

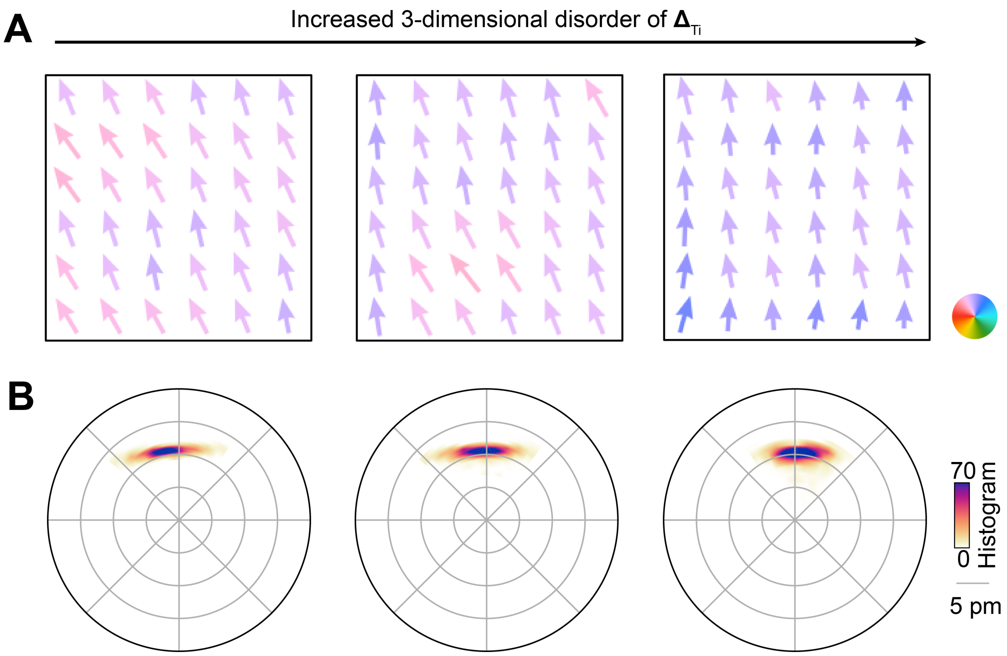

Figure S18: **Effect of 3-dimensional disorder on the projected** ΔTi**.** (A) Projected ΔTi on y-z plane with increased 3D disorder. The color and length of the arrow represents the direction and amplitude. The 3D-structure is acquired from phase-field simulation. (B) Polar histogram of projected ΔTi measured at different 3D disorders. The dataset is 32×32 scale.

REFERENCES AND NOTES

1. M. E. Lines, A. M. Glass, *Principles and Applications of Ferroelectrics and Related Materials* (Oxford Univ. Press, 2001).

2. M. T. Dove, Theory of displacive phase transitions in minerals. *Am. Mineral.* **82**, 213–244 (1997).

3. S. Horiuchi, Y. Tokura, Organic ferroelectrics. *Nat. Mater.* **7**, 357–366 (2008).

4. A. Von Hippel, R. Breckenridge, F. Chesley, L. Tisza, High dielectric constant ceramics. *Ind. Eng. Chem.* **38**, 1097–1109 (1946).

5. V. Garcia, S. Fusil, K. Bouzehouane, S. Enouz-Vedrenne, N. D. Mathur, A. Barthelemy, M. Bibes, Giant tunnel electroresistance for non-destructive readout of ferroelectric states. *Nature* **460**, 81–84 (2009).

6. A. I. Khan, A. Keshavarzi, S. Datta, The future of ferroelectric field-effect transistor technology. *Nat. Electron.* **3**, 588–597 (2020).

7. K.-I. Park, S. Xu, Y. Liu, G.-T. Hwang, S.-J. L. Kang, Z. L. Wang, K. J. Lee, Piezoelectric $BaTiO_3$ thin film nanogenerator on plastic substrates. *Nano Lett.* **10**, 4939–4943 (2010).

8. K.-I. Park, M. Lee, Y. Liu, S. Moon, G.-T. Hwang, G. Zhu, J. E. Kim, S. O. Kim, D. K. Kim, Z. L. Wang, K. J. Lee, Flexible nanocomposite generator made of $BaTiO_3$ nanoparticles and graphitic carbons. *Adv. Mater.* **24**, 2999–3004 (2012).

9. H. Pan, S. Lan, S. Xu, Q. Zhang, H. Yao, Y. Liu, F. Meng, E.-J. Guo, L. Gu, D. Yi, X. R. Wang, H. Huang, J. L. MacManus-Driscoll, L.-Q. Chen, K.-J. Jin, C.-W. Nan, Y.-H. Lin, Ultrahigh energy storage in superparaelectric relaxor ferroelectrics. *Science* **374**, 100–104 (2021).

10. M. Zhang, S. Lan, B. B. Yang, H. Pan, Y. Q. Liu, Q. H. Zhang, J. L. Qi, D. Chen, H. Su, D. Yi, Y. Y. Yang, R. Wei, H. D. Cai, H. J. Han, L. Gu, C.-W. Nan, Y.-H. Lin, Ultrahigh energy storage in high-entropy ceramic capacitors with polymorphic relaxor phase. *Science* **384**, 185–189 (2024).

11. B. W. Wessels, Ferroelectric epitaxial thin films for integrated optics. *Annu. Rev. Mat. Res.* **37**, 659–679 (2007).

12. Y. Cao, S. L. Tan, E. J. H. Cheung, S. Y. Siew, C. Li, Y. Liu, C. S. Tang, M. Lal, G. Chen, K. Dogheche, P. Yang, S. Pennycook, A. T. S. Wee, S. Chua, E. Dogheche, T. Venkatesan, A. Danner, A barium titanate-on-oxide insulator optoelectronics platform. *Adv. Mater.* **33**, 2101128 (2021).

13. F. Jona, G. Shirane, *Ferroelectric Crystals* (Dover Publications, 1993).

14. H. D. Megaw, Temperature changes in the crystal structure of barium titanium oxide. *Proc. R. Soc. London Ser. A Math Phys. Sci.* **189**, 261–283 (1947).

15. W. Cochran, Crystal stability and the theory of ferroelectricity. *Adv. Phys.* **9**, 387–423 (1960).

16. G. Shirane, B. Frazer, V. Minkiewicz, J. Leake, A. Linz, Soft optic modes in barium titanate. *Phys. Rev. Lett.* **19**, 234 (1967).

17. H. Vogt, J. Sanjurjo, G. Rossbroich, Soft-mode spectroscopy in cubic $BaTiO_3$ by hyper-Raman scattering. *Phys. Rev. B* **26**, 5904 (1982).

18. R. Comes, M. Lambert, A. Guinier, The chain structure of $BaTiO_3$ and $KNbO_3$. *Solid State Commun.* **6**, 715–719 (1968).

19. R. Blinc, B. Žekš, Dynamics of order-disorder-type ferroelectrics and anti-ferroelectrics. *Adv. Phys.* **21**, 693–757 (1972).

20. A. Chaves, F. S. Barreto, R. Nogueira, B. Zeks, Thermodynamics of an eight-site order-disorder model for ferroelectrics. *Phys. Rev. B* **13**, 207 (1976).

21. W. Zhong, D. Vanderbilt, K. Rabe, Phase transitions in $BaTiO_3$ from first principles. *Phys. Rev. Lett.* **73**, 1861–1864 (1994).

22. R. Pirc, R. Blinc, Off-center Ti model of barium titanate. *Phys. Rev. B* **70**, 134107 (2004).

23. A. Quittet, M. Lambert, Temperature dependence of the Raman cross section and light absorption in cubic $BaTiO_3$. *Solid State Commun.* **12**, 1053–1055 (1973).

24. B. Ravel, E. Stern, R. Vedrinskii, V. Kraizman, Local structure and the phase transitions of $BaTiO_3$. *Ferroelectrics* **206**, 407–430 (1998).

25. B. Zalar, V. V. Laguta, R. Blinc, NMR evidence for the coexistence of order-disorder and displacive components in barium titanate. *Phys. Rev. Lett.* **90**, 037601 (2003).

26. J. Hlinka, T. Ostapchuk, D. Nuzhnyy, J. Petzelt, P. Kuzel, C. Kadlec, P. Vanek, I. Ponomareva, L. Bellaiche, Coexistence of the phonon and relaxation soft modes in the terahertz dielectric response of tetragonal $BaTiO_3$. *Phys. Rev. Lett.* **101**, 167402 (2008).

27. A. Pugachev, V. Kovalevskii, N. Surovtsev, S. Kojima, S. Prosandeev, I. Raevski, S. Raevskaya, Broken local symmetry in paraelectric $BaTiO_3$ proved by second harmonic generation. *Phys. Rev. Lett.* **108**, 247601 (2012).

28. K. Tsuda, R. Sano, M. Tanaka, Nanoscale local structures of rhombohedral symmetry in the orthorhombic and tetragonal phases of $BaTiO_3$ studied by convergent-beam electron diffraction. *Phys. Rev. B* **86**, 214106 (2012).

29. K. Tsuda, M. Tanaka, Two-dimensional mapping of polarizations of rhombohedral nanostructures in the orthorhombic phase of $KNbO_3$ by the combined use of scanning transmission electron microscopy and convergent-beam electron diffraction. *Appl. Phys. Exp.* **8**, 081501 (2015).

30. K. Tsuda, M. Tanaka, Direct observation of the symmetry breaking of the nanometer-scale local structure in the paraelectric cubic phase of $BaTiO_3$ using convergent-beam electron diffraction. *Appl. Phys. Exp.* **9**, 071501 (2016).

31. Y.-T. Shao, J.-M. Zuo, Nanoscale symmetry fluctuations in ferroelectric barium titanate, $BaTiO_3$. *Acta Crystallogr. B Struct. Sci. Cryst. Eng. Mater.* **73**, 708–714 (2017).

32. R. Comes, M. Lambert, A. Guinier, Désordre linéaire dans les cristaux (cas du silicium, du quartz, et des pérovskites ferroélectriques). *Acta Cryst.* **26**, 244–254 (1970).

33. K. Itoh, L. Zeng, E. Nakamura, N. Mishima, Crystal structure of $BaTiO_3$ in the cubic phase. *Ferroelectrics* **63** (1), 29–37 (1985).

34. E. A. Stern, Character of order-disorder and displacive components in barium titanate. *Phys. Rev. Lett.* **93**, 037601 (2004).

35. B. Zalar, A. Lebar, J. Seliger, R. Blinc, V. V. Laguta, M. Itoh, NMR study of disorder in $BaTiO_3$ and $SrTiO_3$. *Phys. Rev. B* **71**, 064107 (2005).

36. T. Nakatani, A. Yoshiasa, A. Nakatsuka, T. Hiratoko, T. Mashimo, M. Okube, S. Sasaki, Variable-temperature single-crystal X-ray diffraction study of tetragonal and cubic perovskite-type barium titanate phases. *Acta Crystallogr. B.* **72**, 151–159 (2016).

37. S. Ravy, J.-P. Itie, A. Polian, M. Hanfland, High-pressure study of X-ray diffuse scattering in ferroelectric perovskites. *Phys. Rev. Lett.* **99**, 117601 (2007).

38. K. Page, T. Proffen, M. Niederberger, R. Seshadri, Probing local dipoles and ligand structure in $BaTiO_3$ nanoparticles. *Chem. Mater.* **22**, 4386–4391 (2010).

39. M. S. Senn, D. Keen, T. Lucas, J. Hriljac, A. Goodwin, Emergence of long-range order in $BaTiO_3$ from local symmetry-breaking distortions. *Phys. Rev. Lett.* **116**, 207602 (2016).

40. M. Paściak, T. Welberry, J. Kulda, S. Leoni, J. Hlinka, Dynamic displacement disorder of cubic $BaTiO_3$. *Phys. Rev. Lett.* **120**, 167601 (2018).

41. G. Shirane, A. Takeda, Transition energy and volume change at three transitions in barium titanate. *J. Phys. Soc. Jpn.* **7** , 1–4 (1952).

42. J. Chen, L. Hu, J. Deng, X. Xing, Negative thermal expansion in functional materials: Controllable thermal expansion by chemical modifications. *Chem. Soc. Rev.* **44**, 3522–3567 (2015).

43. D. A. Keen, A. L. Goodwin, The crystallography of correlated disorder. *Nature* **521**, 303–309 (2015).

44. L. Gigli, M. Veit, M. Kotiuga, G. Pizzi, N. Marzari, M. Ceriotti, Thermodynamics and dielectric response of $BaTiO_3$ by data-driven modeling. *NPJ Comput. Mater.* **8**, 209 (2022).

45. J. Očenášek, J. Minár, J. Alcalá, Dynamics of lattice disorder in perovskite materials, polarization nanoclusters and ferroelectric domain wall structures. *NPJ Comput. Mater.* **9**, 118 (2023).

46. M. Kotiuga, S. Halilov, B. Kozinsky, M. Fornari, N. Marzari, G. Pizzi, Microscopic picture of paraelectric perovskites from structural prototypes. *Phys. Rev. Res.* **4**, L012042 (2022).

47. T. Malis, S. Cheng, R. Egerton, EELS log-ratio technique for specimen-thickness measurement in the TEM. *J. Electron Microsc. Tech.* **8**, 193–200 (1988).

48. B. H. Savitzky, I. El Baggari, C. B. Clement, E. Waite, B. H. Goodge, D. J. Baek, J. P. Sheckelton, C. Pasco, H. Nair, N. J. Schreiber, J. Hoffman, A. S. Admasu, J. Kim, S.-W. Cheong, A. Bhattacharya, D. G. Schlom, T. M. McQueen, R. Hovden, L. F. Kourkoutis, Image registration of low signal-to-noise cryo-STEM data. *Ultramicroscopy* **191**, 56–65 (2018).

49. X. Shi, J. Wang, J. Xu, X. Cheng, H. Huang, Quantitative investigation of polar nanoregion size effects in relaxor ferroelectrics. *Acta Mater.* **237**, 118147 (2022).

50. T. Yang, B. Wang, J.-M. Hu, L.-Q. Chen, Domain dynamics under ultrafast electric-field pulses. *Phys. Rev. Lett.* **124**, 107601 (2020).

51. M. Nord, P. E. Vullum, I. MacLaren, T. Tybell, R. Holmestad, Atomap: A new software tool for the automated analysis of atomic resolution images using two-dimensional Gaussian fitting. *Adv. Struct. Chem. Imaging* **3**, 9 (2017).

52. C. T. Nelson, B. Winchester, Y. Zhang, S.-J. Kim, A. Melville, C. Adamo, C. M. Folkman, S.-H. Baek, C.-B. Eom, D. G. Schlom, L.-Q. Chen, X. Pan, Spontaneous vortex nanodomain arrays at ferroelectric heterointerfaces. *Nano Lett.* **11**, 828–834 (2011).

53. G. Dong, S. Li, M. Yao, Z. Zhou, Y.-Q. Zhang, X. Han, Z. Luo, J. Yao, B. Peng, Z. Hu, H. Huang, T. Jia, J. Li, W. Ren, Z.-G. Ye, X. Ding, J. Sun, C.-W. Nan, L.-Q. Chen, J. Li, M. Liu, Super-elastic ferroelectric single-crystal membrane with continuous electric dipole rotation. *Science* **366**, 475–479 (2019).

54. Y. Tang, Y. Zhu, X. Ma, W. Wang, Y. Wang, Y. Xu, Z. Zhang, S. Pennycook, Observation of a periodic array of flux-closure quadrants in strained ferroelectric $PbTiO_3$ films. *Science* **348**, 547–551 (2015).

55. P. Gao, A. Kumamoto, R. Ishikawa, N. Lugg, N. Shibata, Y. Ikuhara, Picometer-scale atom position analysis in annular bright-field STEM imaging. *Ultramicroscopy* **184**, 177–187 (2018).

56. J. Madsen, T. Susi, The abTEM code: Transmission electron microscopy from first principles. *Open Res. Eur.* **1**, 24 (2021).
